# RERAS: does age define recovery? The role of patient characteristics and ERAS protocols in robotic colorectal surgery

**DOI:** 10.1007/s11701-026-03426-1

**Published:** 2026-07-20

**Authors:** Mohamad El-Ahmar, S. Abdelhadi, F. Peters, C. Reissfelder, S. Hetjens, B. Mazul, A. Armbruster, F. Spans, M. Dietrich, M. Green, S. Ulmer, M. Ristig, J.-P. Ritz, M. Karg

**Affiliations:** 1 Department of Surgery, Universitätsmedizin Mannheim, Medical Faculty Mannheim, Heidelberg University, Theodor-Kutzer-Ufer 1-3, 68167 Mannheim, Germany; 2 Department of General and Visceral Surgery, Helios Kliniken Schwerin, University Campus Medical School Hamburg, 19055 Schwerin, Germany; 3 Department of Medical Statistics and Biomathematics, Universitätsmedizin Mannheim, Medical Faculty Mannheim, Heidelberg University, Mannheim, Germany

**Keywords:** Robotic Surgery, ERAS, Perioperative Management, Colorectal Resections, Patient-Specifi c Characteristics

## Abstract

Age is frequently considered a major determinant of postoperative recovery after colorectal surgery and is often perceived as a limiting factor for an enhanced recovery after surgery (ERAS^®^) pathway. The integration of ERAS^®^ with a robotic surgical approach (RERAS-concept) may be associated with synergistic effects especially for this group of patients. However, real-world-data evaluating age-related differences in postoperative recovery under RERAS conditions remain limited. This retrospective analysis of a prospectively maintained database included all elective robotic colorectal resections performed between January 2019 and April 2024 under a fully standardized ERAS^®^-protocol. Patients were categorized into younger (≤ 69 years) and older (≥ 70 years) adults. The primary endpoint was the comparison of short-term perioperative outcomes. Secondary endpoints comprised functional recovery parameters and perioperative ERAS^®^ compliance. 344 robotic colorectal resections were analyzed (219 colon and 125 rectal resections). Older adults presented with increased frailty levels across both colon and rectal resections (mFI-5 all p = < 0.0001). In colon resections, this was accompanied by significantly higher preoperative risk profiles, reflected by a greater proportion of ASA III patients (72.1% vs. 40.7%; *p* < 0.0001), while intraoperative characteristics, postoperative morbidity and length of hospital stay were comparable between age groups. Older adults achieved oral pain control earlier (1 vs. 2 days; *p* = 0.0410), reported lower postoperative pain scores from the day of surgery through postoperative day 3 (all *p* < 0.05) and demonstrated greater mobilization on postoperative day 3 (6 vs. 5 h; *p* = 0.0298). In rectal resections, younger patients more frequently received neoadjuvant therapy (59.1% vs. 39%; *p* = 0.0248). Duration of surgery was shorter in older adults (229 vs. 245 min; *p* = 0.0176), while the other endpoints were comparable between cohorts. In this real-world cohort, robotic colorectal surgery within a standardized ERAS^®^ pathway resulted in comparable postoperative recovery across age groups, with statistically significant advantages in pain control and mobilization favoring older adults, supporting age-independent implementation of the RERAS concept.

## Introduction

Hypothetically older patients tend to have more comorbidities with lower physiological reserves, consequently increasing the risk of postoperative complications. On the other side it is thought that younger patients generally experience less postoperative complications. Transitioning this narrative to an evidence-based discussion, urges the importance to acknowledge patient characteristics, particularly in a surgical field. An analysis across the StuDoQ database of the German Society for General and Visceral Surgery from 2017 to 2021 highlights this matter, by reporting a postoperative morbidity rate of 25% following robotic colon- and 34% following rectal resections [1].

Multimodal enhanced recovery after surgery programs like the ERAS^®^ protocols aim to minimize the trauma of hospitalization by improving the perioperative management [2–5]. Therefore, ERAS^®^ protocols are subdivided in over 30 sub steps, including prehabilitation, early postoperative oral nutrition reuptake, early postoperative mobilization as well as the omission of unnecessary catheters and drains [6, 7]. However, one of the core principles of the ERAS^®^ protocols still is represented by the minimal invasive surgical approach [5]. In addition to the traditional laparoscopic approach, robotic colorectal resections increase steadily [1, 3].

The combination principle of robotic colorectal resections with the perioperative treatment of the patients according to the ERAS^®^ protocols demonstrated good results in the past, even in newly implemented ERAS^®^ or robotic centers [3]. Latest studies have even reported promising results of the synergetic effects of that combination principle especially in older patients [8, 9].

Considering the initial hypothesis, these results raise the question, whether the perioperative management according to the ERAS^®^ protocols in combination with a minimal invasive robotic approach leads to similar results in patients with different patient characteristics.

Therefore, in this retrospective analysis of a prospectively maintained database we investigated the effect of the combination between a robotic surgical approach with a multimodal enhanced recovery treatment. The primary endpoint was to compare the patients’ short-term perioperative outcomes within ERAS^®^ protocols, including duration of surgery, conversion rates, postoperative complications according to Clavien-Dindo, anastomotic leakage and reoperation rates, length of hospital stay and readmission rates. Secondary objectives included the evaluation of perioperative ERAS^®^ compliance and functional-recovery-related endpoints.

## Materials and methods

In this retrospective analysis of a prospectively maintained database, all robotic colorectal resections performed between January 2019 and April 2024 using the DaVinci Xi intuitive surgical robot were evaluated. Patients were categorized into two groups based on their age: younger adults (≤ 69 years) and older adults (≥ 70 years). The age cut-off of 70 years was chosen because it lies slightly above the statutory retirement age in Germany (65 years) and therefore represents a group generally regarded as elderly in the national healthcare setting.

All patients enrolled in this study were managed in strict accordance with the Enhanced Recovery After Surgery (ERAS^®^) guidelines [5]. Open surgical procedures, conventional laparoscopic approaches, and emergency colorectal resections were excluded.

Oncologic colorectal resections were performed in compliance with established institutional surgical standards. For colonic malignancies, complete mesocolic excision (CME) was routinely undertaken to facilitate en bloc resection of the mesocolon along intact embryological fascial planes. In patients with rectal cancer, total mesorectal excision (TME) was performed with the objective of achieving optimal locoregional disease control. Surgical indications for benign disease comprised colorectal polyps, diverticular disease (both complicated and uncomplicated), and inflammatory bowel disease.

To ensure a clear understanding of the ERAS^®^ concept, patients underwent a thorough preoperative education conducted by a surgeon from the in-house ERAS^®^ team and a certified ERAS^®^ nurse. For further information each patient received a brochure with key takeaways. In addition, a patient diary was handed to the patients, outlining their in-patient stay step-by-step.

Postoperatively, patients on both cohorts were managed in a standardized manner. All patients were visited daily by the surgical medical team and were continuously guided through their hospital stay by the ERAS^®^ nurse. Postoperative analgesia followed a uniform protocolized pain management regimen across both cohorts and all patients received structured daily physiotherapy throughout hospitalization.

Data documentation was managed by an ERAS^®^ nurse, who entered the information into the ERAS^®^ Interactive Audit System (EIAS), a specialized documentation platform. All data used for this publication were sourced from EIAS.

The colon resections included the following procedures: Ileocecal resection/right hemicolectomy, Sigmoid resection, Left hemicolectomy, Reversal of Hartmann’s situation and oother types of colon resections.

The rectal resections included the following procedures: (Low) Anterior resection of the rectum and Abdominoperineal resections.

Preoperative risk assessment was performed using the American Society of Anestheiologists (ASA) physical status classification. In addition, frailty was evaluated using the modified 5-factor frailty index (mFI-5) to provide an objective and standardized measure of patient vulnerability beyond conventional risk stratification [10, 11].

To assess short-term perioperative outcomes, the following target parameters were collected: Duration of surgery, Conversion rate, Intraoperative blood loss, IMC stay in days, Postoperative complications according to Clavien-Dindo, Anastomotic leakage rate, Reoperation rate, ERAS^®^ related recovery parameters, Compliance to ERAS^®^ Guidelines, Length of hospital stay in days and Readmission rate within the first 30 days.

Complications were classified according to the Clavien-Dindo score [12]. The reoperation rate was limited to two specific causes in EIAS: anastomotic leak and other causes.

All statistical calculations were performed using SAS software, release 9.4 (SAS Institute Inc., Cary, NC, USA). Quantitative approximately normally-distributed parameters are presented by mean values and standard deviations; for skewed data, median and minimum and maximum are given. Qualitative data are described by their absolute and relative frequencies. In order to compare two groups (Robotic, Laparoscopic) regarding qualitative parameters, a Chi-square test or Fisher’s exact test was used, where appropriate. The mean values were compared by two-sample *t*-tests (in the case of normally distributed data) or the Mann-Whitney *U*-test. A p-value of less than 0.05 was considered statistically significant.

## Results

Over the course of the study period, a total of 344 colorectal resections were performed. Among younger adults, 108 colon and 66 rectal resections were undertaken, while in the older adult cohort, 111 colon and 59 rectal resections were performed.

Patient demographics and clinical characteristics are summarized in Tables 1 and 2.

**Table 1:** Patient demographics and clinical characteristics in colon resections

	Younger adults *n* = 108	Older adults *n* = 111	*p*-Value
Age (years)	60 (51–66)	77 (72–82)	–
Gender (Male: Female)	59:49	53:58	0.3084
BMI (kg/m ^2^ )*	28.1 IQR: 24.6–31.9	26.7 IQR: 23.6–29.6	0.2713
ASA-Score* 1 2 3	1 (9.3%) 20 (50%) 44 (40.7%)	2 (1.8%) 29 (26.1%) 80 (72.1%)	< 0.0001
mFI-5 0 1 2 3 4 5	58 34 15 1 – –	9 14 54 26 8 –	**< 0.0001**
Co-morbidities Diabetes Mellitus Severe heart disease Severe Pulmonary disease	23 (21.3%) – 1 (0.9%)	34 (30.6%) 6 (5.4%) 2 (1.8%)	0.1475 0.0292 1.0000
Preoperative WHO performance score* Asymptomatic Symptomatic – ambulant Symptomatic < 50% in bed (in bed during the day)	99 (91.7%) 9 (8.3%) –	98 (88.3%) 11 (9.9%) 2 (1.8%)	0.5878
Diagnosis (Benign: Malignant)	57:51	28:83	**< 0.0001**

BMI (kg/m^2^)*: Body-Mass-Index

ASA-Score*: American Society of Anesthesiologists

mFI-5: Modified 5-factor frailty index

**Table 2:** Patient demographics and clinical characteristics in rectal resections

	Younger adults *n* = 66	Older adults *n* = 59	*p*-Value
Age (years)	60 (54–65)	76 (73–81)	–
Gender (Male: Female)	41:25	17:42	0.2841
BMI (kg/m ^2^ )*	27.2 IQR: 24.6–31.9	26.7 IQR: 23.6–29.6	0.2713
ASA-Score* 1 2 3 4	3 (4.6%) 33 (50%) 29 (43.9%) 1 (1.5%)	1 (1.7%) 17 (28.8%) 39 (66.1%) 2 (3.4%)	0.0561
mFI-5 0 1 2 3 4 5	36 24 5 1 – –	11 18 9 16 5 –	< 0.0001
Co-morbidities Diabetes Mellitus Severe heart disease Severe Pulmonary disease	11 (16.7%) – –	13 (22%) 2 (3.4%) –	0.2986 0.2208 –
Preoperative WHO performance score* Asymptomatic Symptomatic – ambulant Symptomatic < 50% in bed (in bed during the day)	62 (93.9%) 4 (6.1%) –	53 (89.8%) 5 (8.5%) 1 (1.7%)	0.4897
Diagnosis (Benign: Malignant)	7:59	6:53	1.0000
Neoadjuvant Chemo-Radiotherapy	41 (62.1%)	35 (59.3%)	0.0248

BMI (kg/m^2^)*: Body-Mass-Index

ASA-Score*: American Society of Anesthesiologists

mFI-5:Modified 5-factor frailty index

### Specific results for colon resections

A total of 219 robotic colon resections were analyzed, including 108 procedures in younger adults and 111 resections in older adults. Preoperative risk profiles differed significantly between groups, with a substantially higher proportion of ASA 3 patients among older adults (72.1% vs. 40.7%; p = < 0.0001). This difference was further reflected by significantly increased frailty levels in the older cohort, as assessed by the mFI-5, with a median of 1 Point (IQR: 0–2 Points) in younger patients compared to 2 Points (IQR: 1–3 Points) in older patients (p = < 0.0001).

Intraoperative characteristics, including operative time, intraoperative blood loss and conversion rates were comparable between the two cohorts. Overall postoperative morbidity, including complications according to the Clavien-Dindo classification and anastomotic leak and reoperation rates, did not differ significantly.

Within the postoperative ERAS^®^-pathway, several statistically significant differences were observed between age groups. Older adults achieved pain control with oral analgesics earlier than younger patients (1 vs. 2 days; *p* = 0.0410). In addition, patient-reported maximum pain scores, using the visual analog scale (VAS) were consistently lower in older adults on the day of surgery (3 vs. 4; *p* = 0.0012) as well as on postoperative day 1 (3 vs. 4; *p* = 0.0192) and day 3 (1 vs. 2; *p* = 0.0143), whereas no significant difference was observed on postoperative day 2 (2 vs. 2.5; *p* = 0.1328). Moreover, mobilization on postoperative day 3 was significantly higher in older adults (6 h vs. 5 h; *p* = 0.0298), while other functional recovery and ERAS^®^ related parameters as well as and length of hospital stay and Compliance to ERAS^®^ Guidelines were comparable between groups.

However, 30-day readmission rates (4.5% vs. 13%; *p* = 0,0312) was significantly lower in older adults.

Specific results are summarized in Table 3.

**Table 3:** Specific results of the study

	Colon resections *n* = 219			Rectal resections *n* = 125		
	Younger adults *n* = 108	Older aduls *n* = 111	*p*-value	Younger adults *n* = 66	Older aduls *n* = 59	*p*-value
Duration of surgery (in minutes)	167 (143–197)	162 (139–186)	0.2086	245 (213–271)	229 (204–245)	**0.0176**
Conversion rate	5 (4.6%)	-	0.0999	4 (6.1%)	3 (5,1%)	0.6918
Intraoperative blood loss (in mL)	50 (IQR: 30–150)	75 (IQR: 50–200)	0.1392	175 (IQR: 50–300)	150 (IQR: 100–400)	0.6623
Postoperative IMC admission*	5 (4.6%)	11 (9.9%)	0,1937	4 (6.1%)	8 (13.6%)	0.2200
Postoperative complications (Clavien-Dindo) I II IIIa IIIb IVa IVb V	2 (1.9%) 8 (7.4%) 3 (2.8%) 9 (8.3%) 2 (1.9%) - 1 (0.9%)	3 (2.7%) 5 (4.5%) 1 (0.9%) 6 (5.4%) 6 (5.4%) - 1 0.9%)	0.4649	3 (4.5%) 6 (9.1%) 3 (4.5%) 9 13.6%) 2 (3%) 1 (1.5%) 1 (1.5%)	5 (8.5%) 7 (11.9%) 2 (3.4%) 5 (8.5%) 5 (8.5%) - 2 (3.4%)	0.2141
Anastomotic leakage rate	4 (3.7%)	11 (9.9%)	0,1063	10 (15.2%)	5 (8.5%)	0,2834
Reoperation rate	12 (11.1%)	13 (11.7%)	1,0000	14 (21.2%)	12 (20.3%)	1,0000
Intravenous fluid on day of surgery (in mL)	2300 (1725–2875)	2300 (1700–2800)	0.7886	2900 (2300–3400)	2800 (2300–2900)	0.9183
First postoperative bowel movement (in days)	2 (1–3)	2 (1–3)	0.2474	1 (1–2)	1 (1–2)	0.5905
Time until tolerating oral intake of solid food (in days)	1 (1–2)	1 (1–2)	0.6254	1 (1–2)	1 (1–2)	0.3767
Postop. pain control with oral analgesics (in days)	2 (1–3)	1 (1–2)	0.0410	2 (1–3)	1 (1–2)	0.3659
Patient-reported maximum pain (VAS) Day of surgery Day 1 Day 2 Day 3	4 (2–5) 4 (2–5) 2.5 (0–4) 2 (0–3)	3 (0–4) 3 (2–4) 2 (0–3) 1 (0–3)	0.0012 0.0192 0.1328 0.0143	3 (2–4) 4 (2–5) 2 (0–4) 2 (1–3)	3 (1–4) 2 (1–4) 2 (0–3) 2 (0–3)	0.4414 **0.0437** 0.3908 0.3235
Postop. Mobilization Day 1 Day 2 Day 3	4 (3–5) 6 (4–6) 5 (4–6)	4 (4–5) 6 (5–6) 6 (5–7)	0.2861 0.3769 0.0298	4 (2–4) 6 (3–6) 6 (4–6)	4 (2–4) 6 (2–6) 6 (3–6)	0.4333 0.3255 0.3734
Return to ADL (in days)	1.3	1.2	0.3817	1.8	1.7	0.6765
Perioperative ERAS ^®^ -compliance rate	91% (86% – 96%)	92% (88% – 98%)	0.1779	86% (78% – 96%)	85% (76% – 95%)	0.6073
Hospital length of stay (in days)	5 (4–6)	5 (4–6)	0.2838	7 (5–9)	6 (5–11)	0.5999
Readmission rate (Within 30 d postop.) Yes No	14 (13%) 94 (87%)	5 (4.5%) 106 (95.5%)	0,**0312**	12 (18.2%) 54 (81.8%)	6 (10.2%) 53 (89.8%)	0,3075

Postoperative IMC admission*: Postoperative Intermediate-Care-Unit admission

### Specific results for rectal resections

Preoperative oncologic treatment differed significantly between cohorts, with a higher proportion of younger adults receiving neoadjuvant chemotherapy compared with older adults (59.1% vs. 39%; *p* = 0.0248). Although not reaching statistical significance, older adults tended to present with higher preoperative risk profiles, reflected by a greater proportion of ASA 3 patients (66.1% vs. 43,9%; *p* = 0.0561). This trend was paralleled by higher frailty levels in the older cohort, with a median mFI-5 of 1 Point (IQR: 0–1 Points) in younger patients compared to 2 Points (IQR: 1–3 Points) in older patients (p = < 0.0001).

Intraoperative outcomes were largely comparable between both groups. Notably, duration of surgery was significantly shorter in older adults with a median of 229 min (IQR: 204–245 min) compared with 245 min (IQR: 213–271 min) in younger patients (*p* = 0.0176). Intraoperative blood loss and conversion rates did not differ significantly between groups.

Postoperative outcomes were comparable across both cohorts. Overall morbidity, including complications, anastomotic leak- and 30-day readmission rates, showed no significant differences.

Within the ERAS^®^-pathway, older adults reported significantly lower maximum pain scores on postoperative day 1 compared with younger patients (VAS 2 vs. 4; *p* = 0.0437). Other postoperative recovery parameters, including mobilization, return of bowel function, resumption of oral intake and length of hospital stay did not differ significantly between both groups.

Specific results are summarized in Table 3.

## Discussion

Older patients could be characterized as frail and, by extension, presumed to carry an inherently higher perioperative risk when compared with younger individuals endowed with greater physiological reserves and a more robust immune system [13, 14]. Chronological age has consequently often been regarded as a dominant determinant of postoperative outcome [15–17]. This perception has historically fueled the assumption that multimodal enhanced recovery programs such as the ERAS^®^-concept are not applicable to elderly and frail patients, who are believed to require a more conservative and cautious perioperative strategy with delayed mobilization and gradual postoperative rehabilitation. Whether such assumptions are supported by contemporary evidence or rather represent a remnant of outdated surgical dogma, remains a matter of debate.

However, this perception is frequently accompanied by the notion that duration of surgery must be minimized at all costs in older patients, a goal traditionally associated with open surgery or the well-established laparoscopic approach. In contrast, an emerging paradigm challenges this view by proposing that ERAS^®^-principles should be applied independent of chronological age and may, in fact, be particularly beneficial to older and frail patients. From this perspective, early mobilization and early resumption of oral intake for example are considered central components of postoperative recovery rather than risk factors to be avoided [3, 8, 9].

Concurrently, robotic colorectal surgery has evolved from an innovative technology to an integral component of contemporary colorectal surgery within the German healthcare system [1]. Growing evidence suggests that the integration of robotic surgery with standardized ERAS^®^-pathways, the RERAS concept, may result in synergistic benefits by combining technical precision with optimized perioperative care [3, 8]. Yet, despite these advances, it remains unclear whether chronological age continues to exert an independent influence on postoperative outcomes under RERAS conditions.

Against this background, the present study sought to evaluate whether age truly defines recovery under RERAS-conditions or whether postoperative outcomes are predominantly shaped by patient specific characteristics. By providing robust real-world-data on functional recovery parameters that are rarely captured in robotic series, our analysis reflects routine clinical practice and demonstrates that RERAS pathways can compensate for patient heterogeneity independent of chronological age, thereby supporting implementation across all colorectal patients.

In our present study, older adults undergoing robotic colon resections demonstrated postoperative morbidity rates comparable to those of younger patients, despite significantly higher preoperative risk profiles, as reflected by a markedly increased proportion of ASA III patients (72.1% vs. 40.7%; p = < 0.0001). These differences were further substantiated by significantly higher frailty levels in the older cohort, as reflected by the modified 5-factor frailty index, confirming an increased baseline vulnerability beyond conventional ASA classification. Notably, this effect was consistently observed across both colon and rectal resections (all p = < 0.0001). Importantly, older adults reached ERAS^®^-defined recovery milestones at a similar pace to younger patients. When focusing specifically on statistically significant differences, a consistent pattern emerged. All such differences favored the older adult cohort. In colon resections, older patients achieved oral pain control earlier (1 vs. 2 days; *p* = 0.0410) and reported lower maximum pain scores, from the day of surgery (3 vs. 4; *p* = 0.0012) and almost through the entire early postoperative course, including postoperative day 1 (3 vs. 4; *p* = 0.0192) and day 3 (1 vs. 2; *p* = 0.0143). Furthermore, mobilization on postoperative day 3 was significantly higher among older adults (6 h vs. 5 h; *p* = 0.0298), indicating overall preserved functional recovery with comparable postoperative morbidity among both cohorts. In rectal resections, older adults reported significantly lower postoperative pain on postoperative day 1 (VAS 2 vs. 4; *p* = 0.0437). Differences in oncologic pretreatment, including a higher rate of neoadjuvant therapy among younger patients, did not lead to measurable differences in overall functional recovery.

This observation is biologically plausible, as robotic surgery minimizes abdominal wall trauma, preserves pulmonary mechanics and enables stable, low-blood-loss dissection along intact embryological planes [18, 19]. The combination of a reduced inflammatory hit inherent to the robotic minimally invasive approach and the ERAS^®^-driven objective of rapidly restoring patients to their preoperative functional status may be particularly relevant in frail patients with limited physiological reserves. This interaction could explain why chronological age did not translate into impaired recovery parameters, including delayed pain control with oral analgesics, postponed postoperative mobilization, delayed resumption of solid oral intake or prolonged time to first bowel movement. Comparable cohorts reported by Thillainadesan in 2021 and Koh in 2021 similarly demonstrated equivalent morbidity and length of hospital stay among elderly patients managed under ERAS^®^-protocols, supporting the concept that the pathway quality may neutralize age-associated physiological vulnerability [20, 21].

Importantly, age-related differences in prospective pain should also be interpreted in light of physiological changes in pain processing. Lautenbacher demonstrated (2017) in a systematic review and meta-analysis that while subjective pain perception remains largely unchanged with age, pain sensitivity decreases, as reflected by higher thresholds in older adults [22]. Reduced renal opioid clearance in the elderly may further contribute to lower reported pain intensity.

In line with this, van Dijk analyzed more than 11.500 patients from 26 countries and emphasized that postoperative pain outcomes reflect a multifactorial combination of physiological changes with aging and psychological or cultural influences affecting pain perception [23].

From an anesthesiologic perspective, ERAS^®^-compliant intraoperative management further reinforces these effects and appears particularly relevant in the context of robotic surgery. By combining individualized goal-directed fluid therapy, stress-attenuating anesthetic techniques with minimal-residual effects, effective regional anesthesia, active prevention of postoperative nausea and vomiting and strict maintenance of normothermia, perioperative care aims to preserve physiological homeostasis and the surgical stress response. Embedded within a robotic minimally invasive approach, this anesthesiologic framework supports early mobilization and timely resumption of oral intake and may therefore contribute substantially to the age-independent recovery patterns observed under RERAS conditions [24].

The conventional view of age-driven outcome impairment has also been challenged in minimally invasive rectal cancer surgery. Ali reported in 2023 a contemporary multicenter experience demonstrating that older adults undergoing minimally invasive colorectal procedures achieved recovery milestones comparable to younger patients, even in the presence of increased morbidity [25]. Complementing these findings de’Angelis demonstrated in a propensity-score-matched elderly population that robotic colorectal surgery was not associated with inferior intraoperative safety, conversion or reoperation rates when compared to laparoscopy, suggesting that operative technique rather than age per se shapes perioperative trajectories [26].

In rectal resections, age related differences must be interpreted within the oncologic context. In our analysis, younger patients more frequently underwent a neoadjuvant chemoradiotherapy, a factor known to increase operative complexity and potentially prolong duration of surgery. Nevertheless, the approximately 15-minute shorter operative time observed in older patients, although statistically significant, is unlikely to exert clinically meaningful effects on recovery when embedded in a standardized ERAS^®^-pathway. This interpretation is consistent with evidence of comparable postoperative morbidity and recovery parameters in both cohorts, indicating that robotic precision combined with ERAS^®^-principles can compensate for the therapy related complexity. This aligns with the meta-analysis of Reddavid from 2023 and the propensity-matched work of de’Angelis from 2019, both demonstrating age-comparability and safety of robotic surgery in elderly patients, with similar oncologic surgical quality and postoperative morbidity and length of hospital stay [26, 27]. These findings underscore that heterogeneity in baseline oncologic factors does not compromise postoperative recovery when patients are managed within a consistent, protocol-driven pathway.

Notably, reoperation and 30-day readmission rates were lower in the older cohort. Similar observations have been reported in international studies highlighting the feasibility and safety of enhanced recovery care in elderly colorectal surgery. Joris found in 2020 that patients over 70 years benefit from ERAS^®^-programs to an extent comparable with younger patients, without an increase in postoperative morbidity, concluding that age alone should not preclude adherence to structured perioperative pathways [28]. Likewise, Koh confirmed in an age-stratified colorectal cancer cohort that implementation of the ERAS^®^-protocols in elderly patients achieved short-term outcomes equivalent to those of younger patients, underscoring that standardized perioperative management can be maintained independent of age [21]. These data further support the concept that ERAS^®^-pathways may attenuate age associated physiological vulnerability. In addition, in elderly patients, the consistent application of multimodal care elements, including early mobilization and expedited functional recovery, has been associated with promising outcomes [8].

From a health-system standpoint, our data indicate that postoperative allocation to intermediate care occurred more frequently in younger patients than in older adults. This finding implies that advanced chronological age did not translate into increased postoperative resource utilization when patients were managed within a robotic ERAS^®^-pathway. These real-world observations question cost-driven reservation regarding the implementation of robotic ERAS^®^-pathways in elderly populations and offer a relevant practical consideration for institutions contemplating broader adoption of the RERAS concept.

Despite its strengths, this study has several limitations that merit consideration. Although patients were stratified according to chronological age, baseline risk and vulnerability were additionally captured by ASA classification and the mFI-5. However, more comprehensive geriatric assessment, including sarcopenia or cognitive impairment, was not performed and may have enabled a more nuanced evaluation of biological vulnerability and recovery potential. In addition, the analysis was conducted at a single high-volume tertiary center using one robotic platform and a well-established multidisciplinary ERAS^®^-team. Therefore, the external validity of the findings may be limited in settings with differing institutional experience or resource availability. Finally, the study focused exclusively on short-term outcomes. Long-term outcomes including oncologic efficacy and patient reported quality of life have not been included in this study and should be addressed in future multicenter prospective studies.

## Conclusion

In this retrospective study of a prospective maintained database, robotic colorectal resections performed within a perioperative multimodal enhanced recovery program, were associated with largely comparable short-term outcomes across both age groups, despite significantly higher preoperative risk profiles among older adults. Notably, when statistically significant differences were observed, these consistently favored older patients, particularly with regard to postoperative pain control and early mobilization. The synergistic effects of advanced minimally invasive surgery combined with protocolized perioperative management appears to mitigate age-related physiological vulnerability and effectively support functional recovery. These findings provide real-world evidence that the RERAS concept is both feasible and effective, independent of chronological age, supporting its application in all patients undergoing elective colorectal resections. Clinical decision-making should therefore focus on individual patient characteristics and compliance to recovery pathways, rather than by chronological age alone.

## Data Availability

The data that support the findings of this study are available on reasonable request from the corresponding author.
